# Association Between Brain Natriuretic Peptide and Intradialytic Hypotension in Hemodialysis Patients: A Retrospective Study

**DOI:** 10.3390/jcm15187021

**Published:** 2026-09-10

**Authors:** Hui Lu, Shanshan Cheng, Xingzi Liu, Meiling Jin, Fang Sun, Qianmei Sun

**Affiliations:** Department of Nephrology, Beijing Chao-Yang Hospital, Capital Medical University, Beijing 100020, China

**Keywords:** brain natriuretic peptide, intradialytic hypotension, hemodialysis, prediction

## Abstract

**Background:** Intradialytic hypotension (IDH) is a common complication of hemodialysis (HD) and is associated with an increased risk of poor prognosis. This study investigated the factors associated with IDH in HD patients and explored the value of brain natriuretic peptide (BNP) in predicting IDH. **Methods:** Participants were divided into two groups according to the occurrence of IDH. Differences in clinical data between the IDH and no-IDH groups were analyzed, and the influencing factors of IDH were analyzed using logistic regression. A receiver operating characteristic (ROC) curve was applied to identify the optimal critical value of BNP for the occurrence of IDH in HD patients. **Results:** A total of 123 HD patients were included, in which the percentage of IDH was 30.9%. In patients with IDH, the levels of BNP (224.4 vs. 464.7 pg/mL, *p* = 0.001), pre-HD systolic blood pressure (SBP) (153.0 vs. 162.0 mmHg, *p* = 0.002), pre-HD diastolic blood pressure (DBP) (75.7 vs. 84.1 mmHg, *p* = 0.001), post-HD SBP (134.2 vs. 154.2 mmHg, *p* < 0.001) and DBP (72.8 vs. 84.0 mmHg, *p* < 0.001) were significantly lower than those in the non-IDH group, and the level of ejection fraction (66.5 vs. 64.1%, *p* = 0.039) was significantly higher. Univariate analysis showed that BNP [odds ratio (OR) = 0.999, *p* = 0.015], pre-HD SBP (OR = 0.960, *p* = 0.001), and DBP (OR = 0.944, *p* = 0.001) were associated with IDH. Multivariate analysis revealed that lower BNP levels (OR = 0.999, *p* = 0.030), lower pre-HD DBP levels (OR = 0.928, *p* = 0.009), and higher body mass index (BMI) (OR = 1.133, *p* = 0.032) were independently correlated with IDH. BNP had a predictive value for the occurrence of IDH. **Conclusions:** BNP showed predictive value for IDH and may serve as a useful predictor of this complication. Its independent association with IDH supported its potential utility in clinical decision-making.

## 1. Introduction

The number of patients undergoing hemodialysis (HD) has been steadily increasing worldwide over recent decades, driven by the rising prevalence of diabetes, hypertension, and chronic kidney disease [1]. Approximately 4 million people globally are living on kidney replacement therapy (KRT), with HD being the predominant modality, accounting for about 69% of all KRT and 89% of all dialysis treatments [2]. According to the China Kidney Disease Network (CK-NET) report, HD was the primary treatment modality for 92.12% of patients on dialysis in China [3]. Intradialytic hypotension (IDH) is one of the most common complications of HD and is associated with a greater risk of cardiovascular events, low-dose dialysis, vascular access thrombosis, hospitalization, and mortality [4]. Consequently, the identification of reliable predictors of IDH is of paramount clinical importance, as early risk stratification could enable timely preventive interventions and potentially improve long-term patient outcomes.

Brain natriuretic peptide (BNP) is of substantial medical value for the diagnostic evaluation of heart failure and is also a predictor of fluid overload in HD patients, as reported [5,6,7]. In clinical practice, an elevated BNP level typically suggests potential heart disease or excess fluid volume in dialysis patients, leading to efforts to maintain BNP at a lower level. However, the normal reference range of BNP is unclear in patients undergoing dialysis due to incomplete data. The cause of elevated BNP concentration in renal dysfunction is multifactorial, including a counter-regulatory response from the heart to the kidney and diminished passive renal clearance [7]. Blindly lowering BNP levels may lead to hypovolemia. A study revealed that a specific group of patients characterized by a high Δprotidemia and low BNP levels had the highest incidence of IDH [8]. Previous studies have also suggested that higher BNP levels were associated with IDH, as these populations suffered from volume overload [9,10]. These apparently conflicting findings underscore the complexity of the BNP-IDH relationship and highlight the need for further investigation. Moreover, while some studies have explored the association between BNP and IDH, data remain limited and inconsistent, particularly regarding the optimal BNP cut-off value for predicting IDH in the HD population.

Given the scarcity of robust evidence and the conflicting results reported to date, the present study was designed to investigate the relationship between BNP levels and the occurrence of IDH in patients undergoing HD, and to determine the optimal BNP threshold for predicting IDH in this high-risk population.

## 2. Materials and Methods

### 2.1. Study Design and Patients

**Figure 1:**
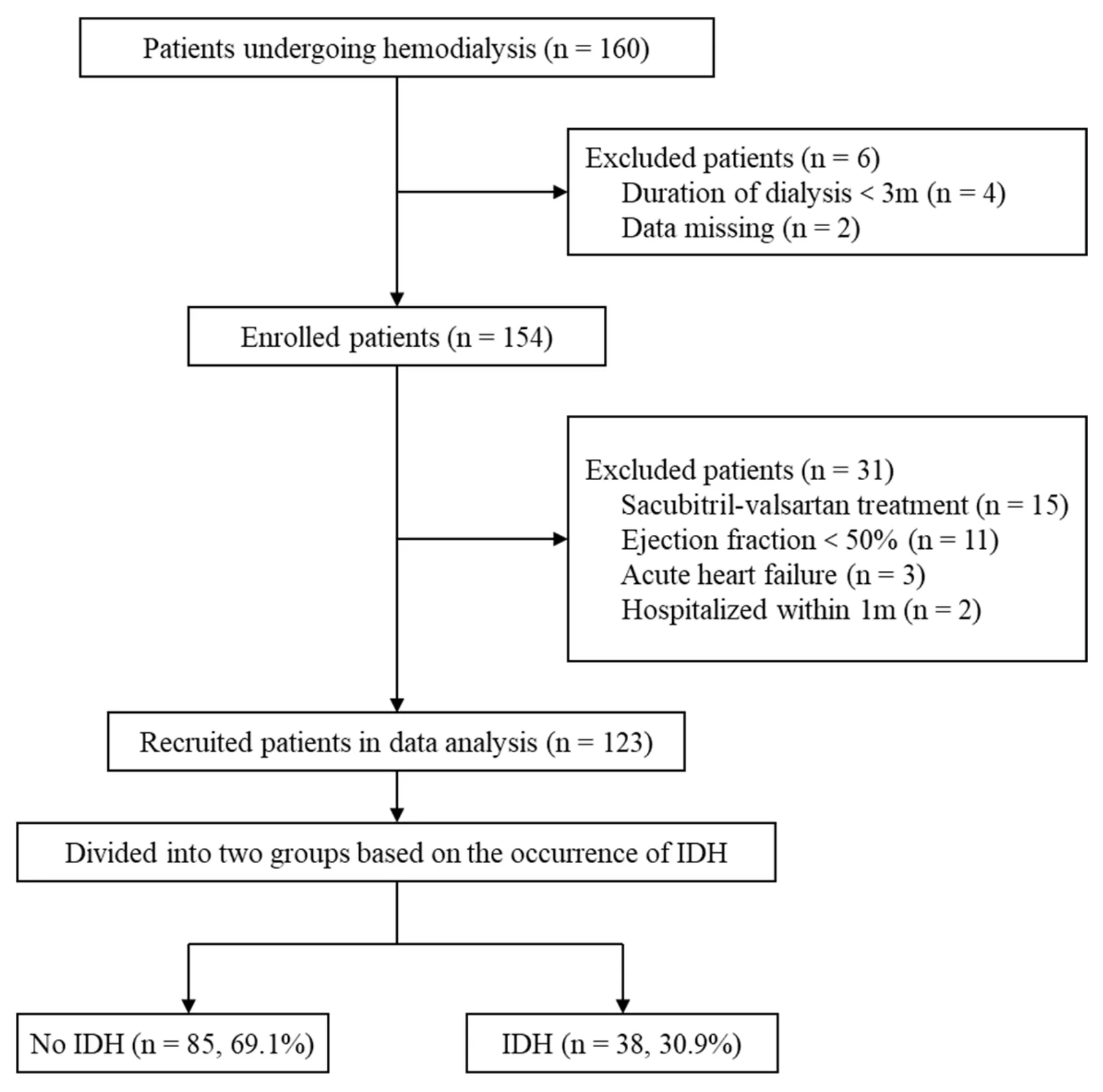
Flow diagram of recruited patients.

This was a single-center observational cohort study in which we retrospectively reviewed the data of end-stage kidney disease (ESKD) patients undergoing HD in the Department of Nephrology at Beijing Chao-Yang Hospital, Capital Medical University, between March and April 2025. The inclusion criteria were age ≥ 18 years, HD treatment duration ≥ 3 months, stable clinical symptoms, and left ventricular ejection fraction (LVEF) ≥ 50%. The exclusion criteria were acute heart failure, acute coronary syndrome, pulmonary embolism, receiving angiotensin receptor-neprilysin inhibitor (ARNI) sacubitril/valsartan treatment, which affects the degradation of NPs, hospitalization within 1 month before study entry, and missing data. Figure 1 shows the flow diagram of patient selection.

This study followed the Declaration of Helsinki and was approved by the ethics committees of Beijing Chao-Yang Hospital, Capital Medical University, Beijing, China (approval number 2026-KE-703). The ethics committees waived the requirement of informed consent due to the observational and retrospective design of the study.

### 2.2. Data Collection

Baseline demographic data of patients, including gender, age, body mass index (BMI), dialysis vintage, etiology of ESKD, past medical histories, and antihypertensive therapy, were collected at the time of entry into the study. All laboratory parameters were measured by automated and standardized methods on the mid-week dialysis day before hemodialysis in March 2025, including complete blood count and blood biochemistry indexes such as serum albumin, serum creatinine, urea, potassium, natrium, calcium, phosphorus, glucose, total cholesterol (TC), high-density lipoprotein cholesterol (HDL-C), low-density lipoprotein cholesterol (LDL-C), triglycerides and B-type natriuretic peptide (BNP). Transthoracic echocardiography was performed, and the left ventricular ejection fraction was measured. Dialysis-related data were collected, including dry weight, dialysis time per treatment, ultrafiltration volume per treatment, and interdialytic weight gain percentage per treatment. Pre-HD and post-HD blood pressure data were collected. The ultrafiltration rate (UR) was calculated as follows: [UR (mL/kg/min) = ultrafiltration volume (mL) ÷ dry weight (kg) ÷ dialysis time (min)]. The interdialytic weight gain percentage (IDWG%) was calculated as IDWG/Weight after last dialysis × 100%. The upper limit of the reference range for BNP was 5000 pg/mL.

### 2.3. IDH

The primary variable in this study was the occurrence of IDH during 12 consecutive HD sessions following the measurement of BNP. Blood pressure was measured by clinical staff during their daily work using an upper arm cuff sphygmomanometer during HD. Blood pressure was measured every 60 min during each dialysis session.

The National Kidney Foundation’s Kidney Disease Outcomes Quality Initiative guidelines define IDH as a decrease in either systolic blood pressure by ≥20 mmHg or mean arterial pressure by ≥10 mmHg, as well as associated symptoms [11,12]. In this study, during the one-month HD treatment period, patients with at least one occurrence of IDH (according to the Kidney Disease Outcomes Quality Initiative) were assigned to the IDH group. Otherwise, they were divided into the non-IDH group.

### 2.4. Statistical Analysis

Statistical analysis was performed using SPSS 23.0 (IBM, New York, NY, USA). Normally distributed variables were described as mean ± standard deviation (SD) and compared using one-way analysis of variance. Data following a non-normal distribution were presented as median [interquartile range (IQR)] and compared using the Mann–Whitney U test. Categorical variables were expressed as amount (percentage) and compared using the chi-square test or Fisher’s exact test when the expected cell counts were small. Logistic regression analysis was performed to assess the associations between baseline characteristics and IDH. Variables with *p* < 0.05 in univariable analysis were entered into the multivariable model, along with clinically relevant confounders regardless of their statistical significance. The relationship between continuous variables and BNP was assessed using the Spearman test. A receiver operating characteristic (ROC) curve was drawn, and the area under the curve (AUC) was used to evaluate the predictive power of BNP for IDH. All probabilities were two-sided, and a *p* < 0.05 was considered statistically significant.

## 3. Results

### 3.1. Baseline Data of the Patients

A total of 1476 dialysis sessions from 123 patients on maintenance dialysis in our dialysis center were analyzed. According to the IDH definition, 62 dialysis sessions experienced IDH, representing 4.2% of the dialysis sessions. Among the 123 patients, 38 (30.9%) were assigned to the IDH group. The clinical characteristics of the enrolled patients were presented in Table 1.

There were 65 (52.8%) male patients; the median age was 60.0 (47.0, 70.0) years, and the median BMI was 22.4 (20.6, 25.8) kg/m^2^. The median dialysis vintage was 77.0 (25.0, 158.0) months. Glomerulonephritis was the leading cause of ESKD in both the IDH and non-IDH groups, followed by diabetes mellitus, hypertension, polycystic kidney disease, and others. Additionally, 18.7%, 6.5%, 34.1%, and 95.1% of patients had a medical history of coronary disease, atrial fibrillation, diabetes mellitus, and hypertension, respectively. There was no difference between the two groups in terms of medical history. Antihypertensive therapy was administered to 84 (68.3%) patients.

Patients with IDH had a higher ejection fraction [66.5 ± 5.5 vs. 64.1 ± 6.1%, *p* = 0.039] than the non-IDH group. The BNP levels [224.4 (73.7, 370.2) vs. 464.7 (221.7, 803.1)] pg/mL, *p* = 0.001, were significantly lower in patients with IDH than in those without IDH. Patients in the IDH group had lower pre-HD systolic blood pressure (SBP) [153.0 (139.8, 161.0) vs. 162.0 (149.5, 169.0) mmHg, *p* = 0.002], pre-HD diastolic blood pressure (DBP) [75.7 ± 11.3 vs. 84.1 ± 12.8 mmHg, *p* = 0.001], post-HD SBP [134.2 ± 20.4 vs. 154.2 ± 17.1 mmHg, *p* < 0.001], and post-HD DBP [72.8 ± 12.1 vs. 84.0 ± 10.3 mmHg, *p* < 0.001] than those in the non-IDH group.

### 3.2. Associations Between Characteristics and IDH

Univariate logistic regression analysis revealed that lower levels of BNP [odds ratio (OR) = 0.999, 95% confidence interval (CI) = 0.997–1.000, *p* = 0.015], pre-HD SBP (OR = 0.960, 95% CI = 0.937–0.985, *p* = 0.001), and pre-HD DBP (OR = 0.944, 95% CI = 0.911–0.978, *p* = 0.001) were associated with IDH (Table 2). Moreover, multivariate logistic regression analysis revealed that higher BMI (OR = 1.133, 95% CI = 1.011–1.270, *p* = 0.032), lower BNP levels (OR = 0.999, 95% CI = 0.997–1.000, *p* = 0.030) and lower pre-HD DBP levels (OR = 0.928, 95% CI = 0.877–0.982, *p* = 0.009) were independent predictors of IDH (Table 3).

### 3.3. Relationship Between Dialysis-Related Parameters and BNP

Correlation analysis revealed that the dialysis vintage (r = −0.179, *p* = 0.048), ejection fraction (r = −0.323, *p* < 0.001), and hemoglobin levels (r = −0.349, *p* < 0.001) were negatively correlated with BNP levels. Pre-HD SBP (r = 0.297, *p* = 0.001) and post-HD SBP (r = 0.498, *p* < 0.001) were significantly positively correlated with BNP levels (Table 4).

### 3.4. Predictive Power of BNP for IDH

**Figure 2:**
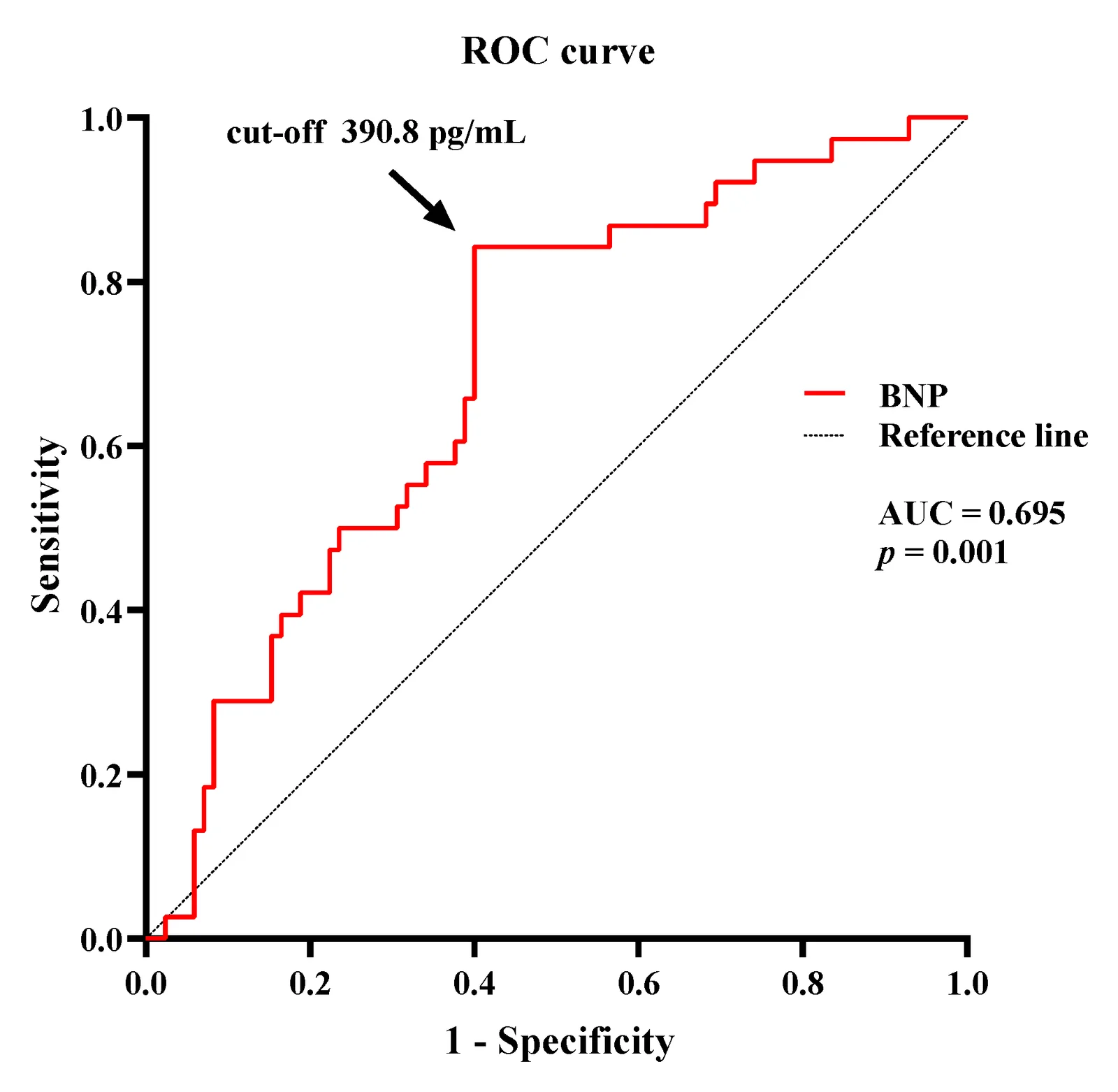
The ROC curve of BNP in assessing IDH. The cut-off value was indicated by a solid arrow.

ROC curve analysis showed that the corresponding AUC of BNP for predicting IDH was 0.695 (95% CI = 0.598–0.792, *p* = 0.001). The optimal cut-off value of BNP for predicting IDH was 390.8 pg/mL (sensitivity = 0.600, specificity = 0.842) (Figure 2).

## 4. Discussion

Our study demonstrated an association between BNP levels and IDH. Multivariate analysis revealed that a lower BNP level was an independent predictor of IDH, suggesting that BNP might serve as a potential biomarker to predict IDH.

IDH is a particularly frequent and concerning symptom in patients undergoing HD. Our observational study found that 38 of 123 patients (30.9%) experienced at least one episode of IDH during the 12 observed dialysis sessions, which was similar to the proportion reported in a previous study [13]. Prior studies have reported that older age, diabetes, and longer dialysis vintage are more frequently observed in patients prone to IDH, but there is still no reliable method to predict it [8,14]. Given that IDH significantly affects quality of life and is linked to higher mortality rates among HD patients, it is important to explore this phenomenon to develop effective therapeutic strategies.

The first driver in the pathophysiology of IDH is the reduction in blood volume caused by ultrafiltration. In our study, the IDH group had lower pre-HD and post-HD blood pressure, as excess volume is an important factor causing hypertension in patients on HD [15,16]. Consequently, we hypothesized that the lower blood pressure observed in the IDH group was attributable to hypohydration. To eliminate the influence of cardiac function on IDH, our inclusion criterion was LVEF ≥ 50%. The results showed that patients with IDH had a significantly higher ejection fraction than the non-IDH group. The plasma BNP levels were significantly lower in the IDH group than in the non-IDH group. In addition, we observed a negative correlation between BNP levels and ejection fraction, which was consistent with previous results [17,18,19].

BNP is affected by many factors, such as age, gender, BMI, atrial fibrillation, cardiac insufficiency, pulmonary embolism, acute coronary syndrome, and anemia [7,20,21]. Many studies have shown a significant correlation between elevated serum BNP levels and overhydration in HD patients, mainly focusing on the upper reference limits for BNP. Fang et al. found a correlation between log-transformed BNP levels and relevant overhydration in HD patients, and those with BNP levels greater than 500 pg/mL had significantly worse survival than patients with lower BNP [22]. Stenberg et al. revealed that BNP correlated positively with overhydration, malnutrition, and inflammation in HD patients, and BNP was best applied for measuring changes in hydration status within an individual over time [23]. However, interpreting these studies is complicated by the difficulty in isolating the individual contributions of volume overload, reduced renal clearance and dialytic removal of the peptides, cardiac structure and function, and adverse events during dialysis to BNP levels [24]. In our study, we aimed to investigate the predictive role of BNP in identifying IDH and to explore a potential threshold that may identify patients at increased risk. There was no statistical difference in age, gender, BMI, anemia index, and history of atrial fibrillation between the IDH and non-IDH groups, and acute heart failure, pulmonary embolism, acute coronary syndrome, and other factors affecting BNP levels were excluded at the time of enrollment. Our research showed that lower BNP levels and pre-HD blood pressure were associated with IDH by univariate logistic regression analysis. BNP levels correlated positively with pre-HD and post-HD SBP. Given that volume status is related to blood pressure in HD patients [25], these findings indicated that BNP might be a potential predictor of hypotension. After adjusting for relevant confounding factors, multivariate analysis revealed that a lower BNP level was an independent predictor of IDH. A BNP level below 390.8 pg/mL was identified as the threshold for predicting IDH in patients undergoing HD.

We also found that a higher BMI was an independent predictor of IDH. Tian et al. revealed that patients with IDH had higher BMI in their study, and it might be that clinical judgment often overestimates the volume status in obese patients, leading to excessive ultrafiltration and volume depletion at the end of a dialysis session [26]. Correlation analysis also showed that BNP was negatively correlated with the dialysis vintage in our study. Dialysis vintage was found to be a significant risk factor for IDH in other studies [13,26]. These results suggested that longer dialysis vintage was associated with lower BNP levels and might increase the risk of IDH.

Accurately assessing the volume status of HD patients in clinical practice is not an easy task. Clinical signs and symptoms are often unreliable, and tests such as bioimpedance and ultrasonographic evaluation of the inferior vena cava are time-consuming and not commonly available [24]. Our study explored the optimal lower threshold of BNP to avoid IDH in HD patients during clinical practice. It would be of great value to increase the frequency of BNP monitoring to prevent IDH in patients who are at risk. Further work to better understand the predictive utility of BNP for IDH and clinical outcomes in patients undergoing dialysis is important.

There were certain limitations that require consideration. First, this research was a retrospective observational cohort study with a relatively small number of patients and short duration, which may lead to bias. Second, the results were based on single-center data without a validation cohort, and external validation is required. Third, we also do not examine dietary sodium intake, dialysate sodium concentration, or sodium fluctuation, which could affect sodium balance and blood pressure stability during dialysis. This should be addressed in future research. Finally, we did not have bioimpedance spectroscopy data to assess fluid status, and further studies on incident patients with body composition monitor (BCM) data are required. Therefore, the results might provide insights and suggest directions for future research. Further well-designed and larger-cohort studies are expected to validate the findings of this study and confirm the predictive performance of BNP for IDH occurrence.

## 5. Conclusions

In conclusion, IDH is common in HD patients and remains a major clinical challenge. We demonstrated that a lower BNP level was associated with a higher risk of IDH, suggesting that BNP might serve as a potentially useful biomarker for identifying patients at increased susceptibility to IDH. In clinical practice, monitoring BNP trends during dialysis sessions could offer a simple and readily available tool to alert clinicians to the risk of hemodynamic instability, thereby enabling more proactive monitoring and early intervention. However, given the observational design of this study, causality cannot be inferred, and volume status was not directly assessed. Therefore, these results should be considered hypothesis-generating, and further prospective studies incorporating objective volume measurements are needed to clarify the potential role of BNP in guiding volume management in HD patients.

## Figures and Tables

**Table 1:** Baseline characteristics of patients with and without IDH.

Characteristic	Total (n = 123)	No IDH (n = 85)	IDH (n = 38)	*p*
Gender [male, n (%)]	65 (52.8%)	44 (51.8%)	21 (55.3%)	0.719
Age (years)	60.0 (47.0, 70.0)	59.0 (45.5, 71.0)	62.0 (53.5, 68.3)	0.632
BMI (kg/m^2^)	22.4 (20.6, 25.8)	22.2 (19.9, 25.5)	23.3 (21.6, 26.8)	0.084
Dialysis vintage (months)	77.0 (25.0, 158.0)	67.0 (23.5, 156.0)	93.0 (35.0, 169.5)	0.235
Ejection fraction (%)	64.8 ± 6.0	64.1 ± 6.1	66.5 ± 5.5	0.039
BNP (pg/mL)	363.2 (145.9, 695.7)	464.7 (221.7, 803.1)	224.4 (73.7, 370.2)	0.001
Hemoglobin (g/L)	112.8 ± 13.6	111.4 ± 14.1	115.8 ± 11.8	0.096
Hematocrit (%)	35.4 ± 4.2	35.0 ± 4.3	36.3 ± 3.6	0.092
RDW-CV (%)	13.9 (13.3, 14.8)	13.9 (13.3, 14.6)	13.9 (13.2, 14.8)	0.707
Albumin (g/L)	39.7 ± 2.7	39.5 ± 2.8	40.2 ± 2.5	0.153
Potassium (mmol/L)	5.1 ± 0.8	5.1 ± 0.7	5.0 ± 0.8	0.870
Natrium (mmol/L)	137.3 ± 2.7	137.3 ± 2.8	137.1 ± 2.5	0.598
Calcium (mmol/L)	2.2 ± 0.2	2.2 ± 0.2	2.2 ± 0.2	0.704
Phosphorus (mmol/L)	1.8 (1.5, 2.2)	2.0 (1.5, 2.3)	1.6 (1.4, 1.9)	0.111
Urea (mmol/L)	23.7 ± 5.8	23.5 ± 6.2	24.2 ± 5.0	0.539
Serum creatinine (μmol/L)	901.5 (727.9, 1129.8)	851.9 (733.4, 1168.9)	915.9 (714.6, 1110.1)	0.969
Glucose (mmol/L)	6.9 (5.7, 9.0)	6.8 (5.8, 8.6)	7.2 (5.6, 9.3)	0.950
TC (mmol/L)	4.1 (3.5, 4.8)	4.1 (3.4, 4.7)	4.4 (3.7, 5.0)	0.172
HDL-C (mmol/L)	1.0 (0.9, 1.2)	1.0 (0.9, 1.2)	1.0 (0.8, 1.2)	0.531
LDL-C (mmol/L)	2.1 ± 0.8	2.1 ± 0.8	2.2 ± 0.8	0.350
Triglyceride (mmol/L)	1.6 (1.1, 2.4)	1.5 (1.1, 2.2)	1.7 (1.4, 2.8)	0.099
Ultrafiltration rate (ml/kg/min)	0.2 ± 0.1	0.2 ± 0.1	0.2 ± 0.1	0.902
Ultrafiltration (L)	2.7 ± 0.9	2.7 ± 0.9	2.8 ± 0.7	0.640
Interdialytic weight gain (%)	4.3 (3.2, 5.1)	4.3 (3.3, 5.2)	4.4 (3.1, 5.0)	0.793
Pre-HD SBP (mmHg)	157.0 (145.0, 168.0)	162.0 (149.5, 169.0)	153.0 (139.8, 161.0)	0.002
Pre-HD DBP (mmHg)	81.5 ± 12.9	84.1 ± 12.8	75.7 ± 11.3	0.001
Post-HD SBP (mmHg)	148.0 ± 20.3	154.2 ± 17.1	134.2 ± 20.4	<0.001
Post-HD DBP (mmHg)	80.5 ± 12.0	84.0 ± 10.3	72.8 ± 12.1	<0.001
Cause of ESKD				
Glomerulonephritis	71 (57.7%)	48 (56.5%)	23 (60.5%)	0.674
Diabetes mellitus	25 (20.3%)	16 (18.8%)	9 (23.7%)	0.536
Hypertension	12 (9.8%)	9 (10.6%)	3 (7.9%)	0.892
Polycystic kidney disease	7 (5.7%)	5 (5.9%)	2 (5.3%)	1.000
Others	8 (6.5%)	7 (8.2%)	1 (2.6%)	0.442
History of coronary disease	23 (18.7%)	12 (14.1%)	11 (28.9%)	0.051
History of atrial fibrillation	8 (6.5%)	6 (7.1%)	2 (5.3%)	1.000
History of diabetes mellitus	42 (34.1%)	28 (32.9%)	14 (36.8%)	0.673
History of hypertension	117 (95.1%)	81 (95.3%)	36 (94.7%)	1.000
Antihypertensive therapy	84 (68.3%)	59 (69.4%)	25 (65.8%)	0.690

Note: Normally distributed variables were shown as mean ± standard deviation (SD). Non-normally distributed variables were presented as median [interquartile range (IQR)]. Categorical variables were shown as number (percentage). IDH, intradialytic hypotension; BMI, body mass index; BNP, brain natriuretic peptide; RDW-CV, red cell volume distribution width-coefficient of variation; TC, total cholesterol; HDL-C, high-density lipoprotein cholesterol; LDL-C, low-density lipoprotein cholesterol; HD, hemodialysis; SBP, systolic blood pressure; DBP, diastolic blood pressure; ESKD, end-stage kidney disease.

**Table 2:** Univariate logistic regression analysis of factors associated with IDH.

Characteristic	Univariable Analysis	
	OR (95% CI)	*p*
Gender [male]	0.869 (0.403, 1.873)	0.720
Age (years)	1.009 (0.980, 1.038)	0.560
BMI (kg/m^2^)	1.066 (0.974, 1.167)	0.165
Dialysis vintage (months)	1.003 (0.998, 1.007)	0.221
BNP (pg/mL)	0.999 (0.997, 1.000)	0.015
Hemoglobin (g/L)	1.026 (0.995, 1.058)	0.098
Hematocrit (%)	1.088 (0.986, 1.201)	0.094
RDW-CV (%)	1.083 (0.800, 1.465)	0.606
Albumin (g/L)	1.114 (0.961, 1.291)	0.153
Potassium (mmol/L)	0.958 (0.574, 1.599)	0.869
Natrium (mmol/L)	0.962 (0.834, 1.110)	0.595
Calcium (mmol/L)	1.599 (0.146, 17.563)	0.701
Phosphorus (mmol/L)	0.574 (0.287, 1.151)	0.118
Urea (mmol/L)	1.021 (0.956, 1.091)	0.536
Serum creatinine (μmol/L)	1.000 (0.998, 1.001)	0.775
Glucose (mmol/L)	1.025 (0.908, 1.157)	0.686
TC (mmol/L)	1.244 (0.880, 1.759)	0.217
HDL-C (mmol/L)	0.545 (0.163, 1.818)	0.323
LDL-C (mmol/L)	1.281 (0.765, 2.145)	0.347
Triglyceride (mmol/L)	1.199 (0.871, 1.651)	0.266
Ultrafiltration (L)	1.115 (0.709, 1.755)	0.637
Interdialytic weight gain (%)	0.923 (0.720, 1.184)	0.529
Pre-HD SBP (mmHg)	0.960 (0.937, 0.985)	0.001
Pre-HD DBP (mmHg)	0.944 (0.911, 0.978)	0.001

Note: IDH, intradialytic hypotension; BMI, body mass index; BNP, brain natriuretic peptide; RDW-CV, red cell volume distribution width-coefficient of variation; TC, total cholesterol; HDL-C, high-density lipoprotein cholesterol; LDL-C, low-density lipoprotein cholesterol; HD, hemodialysis; SBP, systolic blood pressure; DBP, diastolic blood pressure.

**Table 3:** Multivariate logistic regression analysis of factors independently associated with IDH.

Characteristic	Multivariate Analysis	
	OR (95% CI)	*p*
Gender [male]	0.642 (0.242, 1.708)	0.375
Age (years)	0.986 (0.938, 1.035)	0.560
BMI (kg/m^2^)	1.133 (1.011, 1.270)	0.032
Dialysis vintage (months)	1.000 (0.995, 1.006)	0.877
BNP (pg/mL)	0.999 (0.997, 1.000)	0.030
Pre-HD SBP (mmHg)	0.976 (0.946, 1.007)	0.127
Pre-HD DBP (mmHg)	0.928 (0.877, 0.982)	0.009

Note: IDH, intradialytic hypotension; BMI, body mass index; BNP, brain natriuretic peptide; SBP, systolic blood pressure; DBP, diastolic blood pressure.

**Table 4:** Correlation between dialysis-related parameters and BNP.

Characteristic	BNP	
	Correlation Coefficient	*p*
Age (years)	0.083	0.363
BMI (kg/m^2^)	0.003	0.976
Dialysis vintage (months)	−0.179	0.048
Ejection fraction (%)	−0.323	<0.001
Hemoglobin (g/L)	−0.349	<0.001
Ultrafiltration rate (ml/kg/min)	0.005	0.954
Ultrafiltration (L)	−0.034	0.706
Interdialytic weight gain (%)	0.055	0.549
Pre-HD SBP (mmHg)	0.297	0.001
Pre-HD DBP (mmHg)	0.050	0.581
Post-HD SBP (mmHg)	0.498	<0.001
Post-HD DBP (mmHg)	0.149	0.099

Note: IDH, intradialytic hypotension; BNP, brain natriuretic peptide; BMI, body mass index; RDW-CV, red cell volume distribution width-coefficient of variation; TC, total cholesterol; HDL-C, high-density lipoprotein cholesterol; LDL-C, low-density lipoprotein cholesterol; HD, hemodialysis; SBP, systolic blood pressure; DBP, diastolic blood pressure.

## Data Availability

The datasets of the current study are available from the corresponding author on reasonable request.
